# Adrenergic ligands that block oviposition in the cattle tick *Rhipicephalus microplus* affect ovary contraction

**DOI:** 10.1038/srep15109

**Published:** 2015-10-12

**Authors:** Raquel Cossío-Bayúgar, Estefan Miranda-Miranda, Manuel Fernández-Rubalcaba, Verónica Narváez Padilla, Enrique Reynaud

**Affiliations:** 1 Centro Nacional de Investigación Disciplinaria en Parasitología Veterinaria, Instituto Nacional de Investigaciones Forestales Agrícolas y Pecuarias INIFAP. Carr. Fed. Cuernavaca-Cuautla No. 8534, Jiutepec, Morelos, México, 62550; 2 Centro de Investigación en Dinámica Celular, Universidad Autónoma del Estado de Morelos Av. Universidad 1001, Col. Chamilpa, Cuernavaca Morelos, México, 62209; 3 Departamento de Genética del Desarrollo y Fisiología Molecular, Instituto de Biotecnología, Universidad Nacional Autónoma de México, Avenida Universidad, 2001, Apartado Postal 510–3, Cuernavaca, Morelos, México, 62210

## Abstract

The tyraminergic/octopaminergic system is central for the control of arthropod oviposition. Previous works demonstrated that the pharmacological perturbation of this system inhibits oviposition in the cattle tick *Rhipicephalus microplus*. In this work, we describe a physiologically active whole-mount preparation of the contractile tick ovary that allows the quantitative videometrical analysis of ovary contraction in response to different compounds. Eight adrenergic ligands known to inhibit oviposition, including octopamine and tyramine were tested. These compounds exhibited antagonistic effects; octopamine relaxes the ovary preparation while tyramine induces a very strong contraction. The other adrenergic compounds tested were classified as able to contract or relax ovary muscle tissue. Isoprotenerol has a stronger relaxative effect than octopamine. Tyramine induces the biggest contraction observed of all the compounds tested, followed, in descending amount of contraction, by salbutamol, prazosin, epinastine, clonidine and the acaricide amitraz. The effect of these adrenergic ligands on the ovary preparation, explains why these molecules inhibit tick oviposition and suggest a regulatory mechanism for ovary contraction and relaxation during oviposition. Our results also provide a physiological explanation of the egg-laying inhibition effect of amitraz when used on the cattle tick.

*Rhipicephalus microplus* is a worldwide distributed tick in tropical and subtropical environments. This tick is a mayor burden for the livestock industry in these areas because it causes general stress, damages cattle skin, reduces milk and meat production and transmits bovine diseases such as *anaplasmosis* and *babesiosis.* Ticks are usually controlled with chemical acaricides applied on infested cattle. However, abuse in the use of these compounds has selected tick strains that are multi-resistant to most pesticide molecular families^1^. It is important to understand tick physiology in order to identify possible pharmacological targets able to interrupt the biological cycle of livestock pests and develop new control strategies using new anti-acaride molecules. One of the most important acaricides used to control *R microplus* is amitraz, which has been reported to exhibit egg-laying inhibition properties on the cattle tick^2^, but until now no reproductive organ physiological details have been published. It has been proposed that the amitraz target is a biogenic amine receptor, most likely involving the tyraminergic/octopaminergic pathway^2^,^3^. Tyramine and octopamine are two biogenic amines that modulate many aspects of arthropod physiology and behavior. There is extensive literature that documents that the tyraminergic/octopaminergic pathway is the main regulatory system for oviposition control in insects, arachnids, crustaceans and molluscs^4^,^5^,^6^,^7^,^8^. Tyramine was originally considered to be just a precursor for the synthesis of octopamine, however recent literature suggests that it should be considered an independent neurotransmitter as it has a different localization within the insect nervous system, different receptors and transporters and in some cases it can behave as an octopamine antagonist^9^. There are several identified sequences in the genome of *R microplus* G protein coupled biogenic amine receptors that may be potential targets for adrenergic ligands. These include putative: tyramine/octopamine receptor, octopamine α-adrenergic like receptor, octopamine β-adrenergic like receptor, hydroxitriptamine (5HT_1_ and 5HT_7_) receptors, dopamine INDR type receptor, dopamine D1 and D2 receptors, GABAb and muscarinic receptors^10^,^11^. This complex repertoire of biogenic amine receptors has also been identified in other tick species such as the American dog tick *Dermacentor variabilis*^12^. In previous works it has been demonstrated that α and β adrenergic ligands are able to inhibit oviposition in *R microplus in vivo*^2^,^13^. The relevance of adrenergic signaling pathways in arthropod oviposition is supported by the fact that there is one octopamine receptor that shows similarities with the mammalian α-adrenergic receptor (Oamb) and several others that share significant similarities with β-adrenergic receptors. Both the Oamb and octβ2R receptor are indispensable for oviposition in *Drosophila*^5^,^14^,^15^. The identification of the orthologous octβ2R octopamine receptor in *R microplus* as well as the inhibition of oviposition by adrenergic ligands strongly suggest that similar pathways are involved in ticks^5^,^10^,^16^,^17^. Arthropod ovaries require the combined effect of contraction and relaxation in order to push mature eggs into the oviducts so they can be fertilized and oviposited^17^,^18^. Ovary contraction and relaxation can be observed and measured using videometric techniques to document changes of the area (A) that the ovary covers^17^. In order to measure ovary contraction we developed a physiologically active tick ovary preparation and an image processing protocol that can be used to evaluate the effect of different substances on this organ.

The objective of this work is to demonstrate that adrenergic ligands have a direct effect on the contraction of the *R microplus* ovary muscle.

## Results

### Adrenergic ligands have an effect on ovary contraction

**Figure 1:**
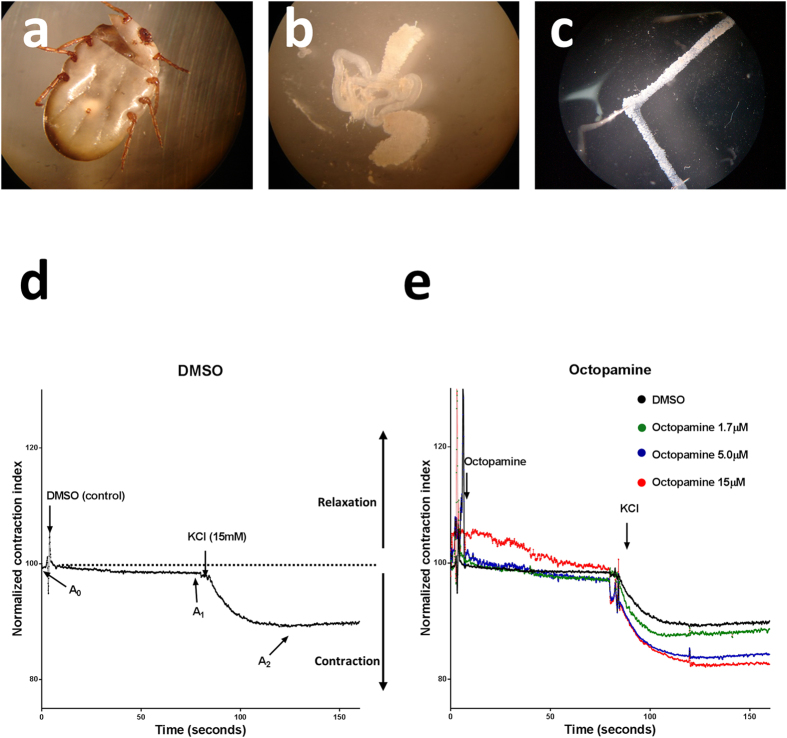
Physiologically active whole-mount preparation of the contractile tick ovary. (**a**) Dissection procedure for the extraction and mount of the contractile tick ovary, ticks are transversally cut between the first and second leg pairs. (**b**) Internal organs are extruded and **(c**) the ovary is pinned in a Sylgard surface. (**d**) Physiological paradigm for the study of ovary contraction A_0_ corresponds to the ovary basal tone in the presence of Ca^2+^ 2 mM. A_1_ corresponds to the area of the ovary after 80 seconds of incubation with the substance to be tested, and reflects the effect on muscle tone for the substance tested. In this case the vehicle (0.05% DMSO in complete Jan & Jan solution) was used as control. A_2_ corresponds to maximal contraction after de addition of 15 mM KCl. A normalized contraction index (NCI) smaller than 100 means that the tissue has an increase in tone or contraction correspondingly, a NCI bigger than 100 means that the tissue relaxes or loses tone. All traces (time series) represent the averaged response of six preparations. All experimental conditions tested were compared to this control. (**e**) Octopamine dose response, three different concentrations were tested (15 μM, 5 μM, 1.7 μM). At 1.7 μM the muscle tone (A_1_) was not significantly affected. All other conditions were significantly different (*P* ≤ 0.0001).

To stablish a standardized paradigm for the evaluation of molecules that have the potential to affect ovary contraction, physiologically active preparations of tick ovaries were prepared (Fig. 1a–c) and a series of control contraction curves were performed. To evaluate the response, we used the normalized contraction index (NCI) which is defined as the area of the ovary at any moment divided by its initial area (A_0_) multiplied by 100. If the tissue has a normalized contraction index smaller than 100 it means that it has increased its tone or amount of contraction. If the tissue contraction index is bigger than 100, it means that the tissue relaxes or loses tone. In the control experiments, ovaries were only exposed to the vehicle for the substances tested (0.05% DMSO in complete Jan & Jan solution). Without any treatment, ovary muscle tone has a slight tendency to keep augmenting after Ca^2+^ as observed in the area delimited by the A_0_ and A_1_ marks (Fig. 1d). Addition of 15 mM KCl after 80 seconds induces a strong contraction as shown by the A_2_ mark (Fig. 1d). Previous data^13^ suggested that 5 mM octopamine would elicit a strong response. As proof of principle, we performed a dose response test using three different concentrations of this molecule (15 μM, 5 μM and 1.7 μM). The response of the ovaries was proportional to the concentration and, compared to the control as well as in between them, they were significantly different (Fig. 1e, Table 1).

**Figure 2:**
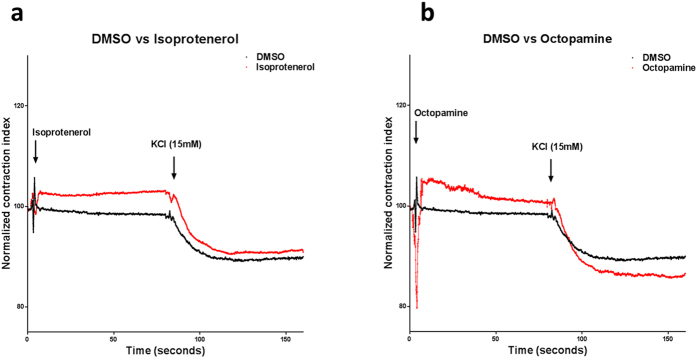
Isoprotenerol and octopamine diminish ovary muscle tone. (**a**) Isoprotenerol strongly reduces basal muscle tone and inhibits maximal muscle contraction. (**b**) Octopamine strongly reduces basal muscle tone but enhances maximal muscle contraction. In both cases Chi-squared test and Dunnet´s multiple comparison test compared to control was *P* ≤ 0.0001, n = 6.

Our data shows that isoprotenerol is the only substance tested that reduces both muscle tone and maximal ovary contraction. Upon isoprotenerol addition, there is a rapid and significant reduction of basal muscle tone which is consistent with isoprotenerol´s known selective β_1_ and β_2_ agonist activity. Isoprotenerol also affects maximal ovary contraction since treated ovaries do not contract as much as control ones, however there is probably a compensation effect since the change between A_1_ and A_2_ is significantly bigger in the treated ones (Δ isoprotenerol A_2_ − A_1_ = −11.12 ± 2.75 vs ΔDMSO A_2_ − A_1_ = −9.13 ± 2.68, P ≤ 0.0001) (Fig. 2a, Table 2). Octopamine, significantly reduced muscle tone (octopamine A_1_ = 102.06 ± 4.81 vs DMSO A_1_ = 98.96 ± 2.68 P ≤  = .0001) and increased maximal ovary contraction (octopamine A_2_ = 86.62 ± 4.81 vs DMSO A_2_ = 89.65 ± 2.68 P ≤  = .0001). This bimodal effect is probably because octopamine is an α and β adrenergic agonist and these two signal transduction pathways are antagonistic. The change between A_1_ and A_2_ in octopamine treated ovaries is also bigger (Δ octopamine A_2_ − A_1_ = −15.43 ± 4.81 vs ΔDMSO A_2_ − A_1_ = −9.13 ± 2.68, P ≤ 0.0001) (Fig. 2b, Table 2).

**Figure 3:**
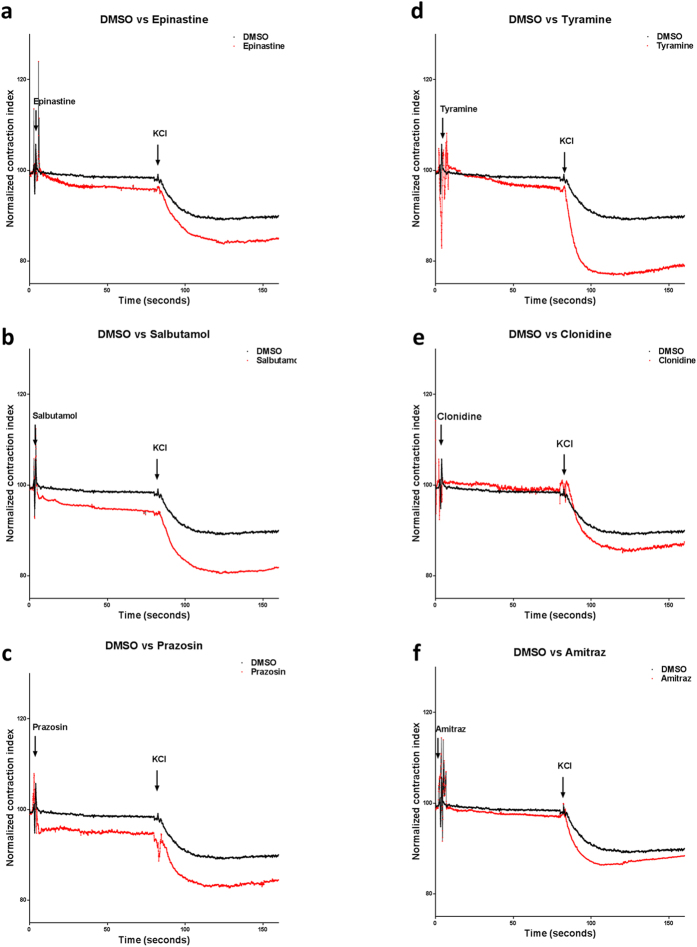
a-adrenergic ligands augment muscle contraction. (**a–c**) Epinastine, salbutamol and prazosin augment muscle tone and maximal muscle contraction. (**d**) Tyramine mildly augments muscle tone and is the strongest inducer of maximal muscle contraction. (**e,f**) Clonidine and amitraz do not have an effect on muscle tone but induce a stronger maximal muscle contraction. In all cases Chi-squared test and Dunnet´s multiple comparison test compared to control was *P* ≤ 0.0001, n = 6.

Epinastine, salbutamol and prazosin had a strong contractile effect on muscle tone (epinastine A_1_ = 97.31 ± 1.73, salbutamol A_1_ = 95.98 ± 3.02, prazosin A_1_ = 95.75 ± 3.38 vs DMSO A_1_ = 98.96 ± 2.68 P ≤ = 0.0001) (Fig. 3a–c, Table 2). Tyramine had a smaller but significant contractile effect on muscle tone (tyramine A_1_ = 98.08 ± 2.94 vs DMSO A_1_ = 98.96 ± 2.68 P ≤ = .0001) while clonidine and amitraz only affected it marginally (clonidine A_1_ = 99.73 ± 3.75, amitraz A_1_ =  98.33 ± 5.05) (Fig. 3d–f, Table 2). Maximal ovary contraction was most enhanced by the antihistamine epinastine, the β_2_ agonist salbutamol, the α adrenergic blocker prazosin and tyramine, which was the strongest inductor of maximal ovary contraction (epinastine A_2_ = 84.74 ± 1.73, salbutamol A_2_ = 81.29 ± 3.02, tyramine A_2_ = 77.82 ± 2.94 vs DMSO A_2_ = 89.65 ± 2.68 P ≤ = .0001) (Fig. 3a–d, Table 2). Clonidine and amitraz were less potent but still induced a significant increase in A_2_ (clonidine A_2_ = 86.35 ± 3.75, amitraz A_2_ = 87.31 ± 5.05, P ≤ 0.0001) (Fig. 3e,f, Table 2).

## Discussion

The objective of this work is to determine if the inhibitory effect on tick oviposition of some adrenergic substances correlates with changes in the contractibility of the ovary. These data contribute to the understanding of the role of adrenergic ligands on the physiology of oviposition in the cattle tick, which eventually could lead to new alternatives for the control of this burden of the cattle industry. Our model could also be adapted for the understanding of the oviposition physiology of other pests such as mosquitoes and flies and the development of new strategies for their control. Several physiological functions of invertebrates are regulated by biogenic amine receptors that activate α and β-like adrenergic pathways. These two pathways are antagonistic in many relevant physiological processes, including oviposition, and their balanced activity is central for the correct function of the organism^4^,^13^,^14^,^16^,^17^,^18^,^19^,^20^,^21^.

Octopamine and Isoprotenerol should be classified as relaxants because they reduce ovary muscle tone. Isoprotenerol was by far the most potent relaxant because it not only reduced ovary muscle tone, but it also strongly diminished final ovary muscle contraction (Fig. 2a) this is in accordance with isoprotenerol´s classic smooth muscle relaxant effect on mammalian systems^22^. Octopamine, one of the arthropod endogenous ligands, reduced basal ovary muscle tone probably by stimulating the production of cAMP as expected if it is inducing a β like response^17^. Interestingly, it also induced contraction after depolarization (Fig. 2b). These data suggest that octopamine may also have an α adrenergic activity in the tick as predicted by the presence of an octopamine α-adrenergic-receptor-like gene in its genome^10^.

Epinastine, salbutamol, prazosin, tyramine, clonidine and amitraz also induce contraction. Epinastine has been reported as a specific antagonist of the β-like-adrenergic activity of octopamine^23^ and correspondingly it strongly incremented both ovary muscle tone and maximal ovary contraction (Fig. 3a), therefore supporting the hypothesis that the β-like activity of octopamine plays a central role in tick ovary relaxation as has been demonstrated in *Drosophila*^17^. Salbutamol and prazosin also augmented muscle tone and incremented maximal ovary contraction (Fig. 3b,c). In humans, salbutamol is a β agonist and prazosin is an α blocker and both are used to relax smooth muscle^24^,^25^. The seemingly opposed effect of these substances observed on tick smooth muscle could be explained by a hyper-activation of the tick β-like receptors. This effect has been observed in human bronchial hyper-responsiveness. It has been documented to be caused either by differential isomer sensitivity or by inducing self-negative regulation (of the β-adrenergic receptor) therefore overriding β-like signaling by receptor desensitization. Ligands could also be promiscuously activating the α-like signaling pathway that normally induces smooth muscle contraction^26^,^27^,^28^. This phenomenon suggests that these molecules do bind to tick adrenergic like receptors but may reflect evolutionary divergence between mammals and arachnids signal transduction pathways; alternatively, these differences can also be explained if tick receptors have different ligand affinities and therefore the strength of signal transduction would be different. This evolutionary divergence in ligand affinity and signal transductions pathways should be studied in the future but are beyond the scope of this work. Tyramine, the other monoamine endogenous ligand, is the most potent tick ovipositon inhibitor that we have tested^13^. Tyramine mildly augments muscle tone and it is also the most potent inducer of maximal contraction tested, strongly suggesting that it mainly activates the α-adrenergic like^10^ pathway in this organism, thus supporting the idea that in some cases, it can behave as an octopamine antagonist and that it should be considered an independent neurotransmitter^9^ (Fig. 3d). Amitraz and clonidine, two of the most potent oviposition inhibitors tested^13^, had minimal effect on muscle tone but induced a significantly bigger maximal contraction than control (DMSO)(Fig. 3e,f). Amitraz is a very potent inhibitor of tick oviposition, however it has been proposed that a metabolite derived from amitraz is actually the biologically active acaricide^1^,^19^,^29^. The relatively low effect on ovary contraction in our organ preparation can therefore be explained for the lack of either time to process amitraz or the absence of metabolic tissue such as the fatbody or the hepatopancreas needed to produce the biologically active metabolite.

In conclusion, the observed effects of these molecules provide a possible mechanism for the inhibition of oviposition by adrenergic ligands, and our oviduct preparation is physiologically relevant therefore providing an expedite system for the identification of substances that affect fertility in arthropods.

## Methods

### Tick strain

“Media joya” *R microplus* acaricide susceptible reference strain (Su) was used. This strain has been cultured under laboratory conditions for many generations and was obtained from the Centro Nacional de Investigación Disciplinaria en Parasitología Veterinaria (CENID-PAVET-INIFAP) at Jiutepec, Morelos, México. Ticks were obtained by infesting a bovine with 1 × 10^4^ fifteen days old larval ticks. Pre-engorged ticks were collected for analysis twenty days after infestation.

### Adrenergic ligands

Isoprotenerol, salbutamol, prazosin, clonidine were obtained from an adrenergic ligand library commercialized by Biomol International (cat. No. 2811 Plymouth Meeting, PA, USA). Epinastine, tyramine, amitraz, and octopamine were obtained from Sigma chemical company, St. Louis, MO, USA.

### Physiologically active whole-mount preparation of contractile ovary and ovary response to adrenergic compound paradigm

Ovaries were extracted from the pre-engorged females (19 to 20 days after infestation) using the following procedure: female ticks were washed with distilled water to remove any extraneous debris. A transversal cut was performed between the first and second leg pairs removing the whole anterior area. Internal organs, were extruded in Jan & Jan^30^ solution (NaCl 128 mM, KCl 2 mM, 4 mM MgCl_2_, Sucrose 36 mM, HEPES 5 mM pH = 7.3) without Ca^2+^ to avoid neurotransmitter depletion during ovary dissection and preparation (Fig. 1a–c). Ovaries were extended avoiding excess of tension and pinned on a Sylgard plate in a homemade perfusion chamber using stainless steel Austerliz Insect Pins (minutiens 0.1 mm Fine Science tools) and the rest of the reproductive and internal organs were discarded. Once ovaries were mounted on the Sylgard plate, the Jan & Jan Ca^2+^ free solution was substituted by complete Jan & Jan solution (NaCl 128 mM, KCl 2 mM, 4 mM MgCl_2_, Sucrose 36 mM, 2 mM Ca^2+^, HEPES 5 mM pH = 7.3). The Ca^2+^ addition induced an increase in the ovary muscle tone thus allowing assessment of tissue integrity and defining ovary initial area (A_0_) (Fig. 1c). The normalized contraction index (NCI), is defined as the area of the ovary at any moment divided by A_0_ multiplied by 100. A NCI smaller than 100 means that the tissue has an increase in tone or contraction. A NCI bigger than 100 means that the tissue relaxes or loses tone. The effect of the substance tested on muscle tone (A_1_) is measured for 70 s of exposure to treatment and averaged. Depolarization with 15 mM KCl induced in all conditions, a strong contraction of the tissue. Final or maximal ovary contraction (A_2_) is defined as the average of the last 70 seconds after the addition of KCl (Fig. 1d). In all experiments, the sample was perfused with Jan & Jan solution supplemented with 5 μM of the adrenergic ligand tested, except for the octopamine dose response experiment where the different concentrations were 15 μM, 5 μM and 1.7 μM. All molecules were dissolved in DMSO and its final concentration was in all experiments 0.05%. The response of at least six ovaries was measured per substance.

### Videometrical analysis of the contractile ovary

Ovary muscle contraction was recorded with a Nikon SMZ100 dissection microscope equipped with a digital camera and recorded in an eight bit grey scale at 15 frames per second using a resolution of 1024 × 1024 pixels per frame and at 80X final magnification. Images were analyzed using the ImageJ program. A section that covered at least 25% each ovary area was defined by placing a mask to define the region of interest (ROI). Images were processed using the following protocol: 1) Automatic subtract background (50 pixel rolling ball radius). 2) Automatic threshold was applied in all frames. 3) Make binary. 4) Analyze particles of 100 to infinity pixel area in order to discard from measurements small debris and other artifacts. 5) Particle pixel area values for each time series were exported to excel files for further analysis.

For each ovary, the ovary area average of the first 15 frames before molecule addition was used as the initial condition (A_0_). Ovary contraction index is defined as frame area value (A_f_) normalized with A_0_ using the following formula:


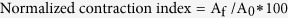


### Statistical analysis

For each frame (data point) the normalized contraction index was averaged between samples of the same treatment (n = 6) and its standard deviation was calculated. Using this data, experimental times series were compared to control (0.05% DMSO in complete Jan & Jan solution) using the Chi-square test to determine if there was a significant difference between normalized contraction index along the whole time series. If the *P* value of the Chi-squared test compared to control was ≤0.05 it is considered that a significant difference exists between experimental conditions and control^31^,^32^. Experimental time series were also analyzed using ANOVA with Dunnett´s multiple comparison test, separate analysis was performed for the muscle tone component of the time series, the contraction component of the time series and for the whole experimental time series *P* values ≤ 0.01 were considered significative. Experimental measurements are included in Dataset 1 and statistical test details are included in supplementary tables S1 to S4.

## Additional Information

**How to cite this article**: Cossío-Bayúgar, R. *et al.* Adrenergic ligands that block oviposition in the cattle tick *Rhipicephalus microplus* affect ovary contraction. *Sci. Rep.* **5**, 15109; doi: 10.1038/srep15109 (2015).

## Supplementary Material

Supplementary Information

Supplementary Information

## Figures and Tables

**Table 1:** ANOVA Tukey's multiple comparisons test of octopamine’s dose response.

	Mean Diff.	95% CI of diff.	Significant?	Summary
Octopamine 5 μM vs. Octopamine 1.7 μM	−1.43	−1.94 to −0.916	Yes	****
Octopamine 5 μM vs. Octopamine15 μM	−0.886	−1.40 to −0.374	Yes	***
Octopamine 1.7 μM vs. Octopamine15 μM	0.542	0.0297 to 1.05	Yes	*

In all cases: n = 6 *P ≤0.01 ***P ≤ 0.001 ****P ≤ 0.0001, alpha 0.05.

**Table 2:** Curve Parameters.

Substance	A_1_(tone)	A_2_(contraction)	DA_2_ – A_1_	
DMSO	98.96 ± 2.68	89.65 ± 2.68	−9.13 ± 2.68	
isoprotenerol	102.34 ± 2.75	91.14 ± 2.75	−11.12 ± 2.75	
tyramine	98.08 ± 2.94	77.82 ± 2.94	−20.03 ± 2.94	
salbutamol	95.98 ± 3.02	81.29 ± 3.02	−14.48 ± 3.02	
epinastine	97.31 ± 1.73	84.74 ± 1.73	−12.39 ± 1.73	
clonidine	99.73 ± 3.75	86.35 ± 3.75	−13.38 ± 3.75	
prazosin	95.75 ± 3.38	83.64 ± 3.38	−11.97 ± 3.38	
amitraz	98.33 ± 5.05	87.31 ± 5.05	−11.02 ± 5.05	
octopamine	102.06 ± 4.81	86.62 ± 4.81	−15.43 ± 4.81	
Statistical significance of Tone (A_1_), Contraction (A_2_) and Whole Curves				
**ANOVA Dunnett's multiple comparisons test**	**Mean Diff.**	**95% CI of diff.**	**Significant?**	**Summary**
Tone (A_1_)				
DMSO vs. Isoprotenerol	−3.55	−3.77 to −3.32	Yes	****
DMSO vs. Tyramine	1.15	0.925 to 1.37	Yes	****
DMSO vs. Salbutamol	3.22	3.00 to 3.44	Yes	****
DMSO vs. Epinastine	1.83	1.61 to 2.06	Yes	****
DMSO vs. Clonidine	−0.806	−1.03 to −0.584	Yes	****
DMSO vs. Prazosin	3.32	3.10 to 3.54	Yes	****
DMSO vs. Amitraz	0.624	0.402 to 0.846	Yes	****
DMSO vs. Octopamine 5 μM	−0.522	−0.744 to −0.300	Yes	****
DMSO vs. Octopamine 1.7 μM	0.102	−0.120 to 0.324	No	Ns
DMSO vs. Octopamine15 μM	−3.21	−3.43 to −2.99	Yes	****
Contraction (A_2_)				
DMSO vs. Isoprotenerol	−1.99	−2.38 to −1.61	Yes	****
DMSO vs. Tyramine	11.2	10.8 to 11.5	Yes	****
DMSO vs. Salbutamol	7.75	7.37 to 8.14	Yes	****
DMSO vs. Epinastine	4.42	4.03 to 4.80	Yes	****
DMSO vs. Clonidine	2.55	2.17 to 2.94	Yes	****
DMSO vs. Prazosin	5.85	5.47 to 6.24	Yes	****
DMSO vs. Amitraz	2.45	2.07 to 2.84	Yes	****
DMSO vs. Octopamine 5 μM	5.17	4.79 to 5.56	Yes	****
DMSO vs. Octopamine 1.7 μM	1.7	1.31 to 2.08	Yes	****
DMSO vs. Octopamine15 μM	6.09	5.70 to 6.47	Yes	****
Whole Curve (tone and contraction phases)				
DMSO vs. Isoprotenerol	−2.77	−3.30 to −2.24	Yes	****
DMSO vs. Tyramine	6.16	5.63 to 6.69	Yes	****
DMSO vs. Salbutamol	5.49	4.96 to 6.02	Yes	****
DMSO vs. Epinastine	3.13	2.60 to 3.66	Yes	****
DMSO vs. Clonidine	0.874	0.345 to 1.40	Yes	****
DMSO vs. Prazosin	4.59	4.06 to 5.12	Yes	****
DMSO vs. Amitraz	1.54	1.01 to 2.07	Yes	****
DMSO vs. Octopamine 5 μM	2.33	1.80 to 2.86	Yes	****
DMSO vs. Octopamine 1.7 μM	0.901	0.371 to 1.43	Yes	****
DMSO vs. Octopamine15 μM	1.44	0.913 to 1.97	Yes	****

In all cases: n = 6 *P ≤ 0.01 ***P ≤ 0.001 ****P ≤ 0.0001, alpha 0.05.
