# Supplementary material for: Adrenergic ligands that block oviposition in the cattle tick Rhipicephalus microplus affect ovary contraction
Source: Sci Rep. 2015 Oct 12;5:15109. doi: 10.1038/srep15109 (PMC4601016; doi:10.1038/srep15109)
Supplement: Supplementary Information [file srep15109-s1.pdf]

Adrenergic ligands that block oviposition in the cattle tick *Rhipicephalus microplus* affect ovary contraction

Raquel Cossío-Bayúgar , Estefan Miranda-Miranda , Manuel Fernández-Rubalcaba , Verónica Narváez-Padilla and Enrique Reynaud

S1-ANOVA whole curve

Whole curve								
ANOVA summary								
F	353							
P value	< 0.0001							
P value summary	****							
Are differences among means statistically significant? (P < 0.05)	Yes							
R square	0.118							
Brown-Forsythe test								
F (DFn, DFd)	238 (10, 26389)							
P value	< 0.0001							
P value summary	****							
Significantly different standard deviations? (P < 0.05)	Yes							
Bartlett's test								
Bartlett's statistic (corrected)	2876							
P value	< 0.0001							
P value summary	****							
Significantly different standard deviations? (P < 0.05)	Yes							
ANOVA table	SS	DF	MS	F (DFn, DFd)	P value			

Treatment (between columns)	160783	10	16078	F (10, 26389) = 353	P < 0.0001			
Residual (within columns)	1200000	26389	45.5					
Total	1360000	26399						
Data summary								
Number of treatments (columns)	11							
Number of values (total)	26400							
Number of families	1							
Number of comparisons per family	10							
Alpha	0.05							
Dunnett's multiple comparisons test	Mean Diff.	95% CI of diff.	Significant?	Summary				
DMSO vs. Isoprotenerol	-2.77	-3.30 to -2.24	Yes	****				
DMSO vs. Tyramine	6.16	5.63 to 6.69	Yes	****				
DMSO vs. Salbutamol	5.49	4.96 to 6.02	Yes	****				
DMSO vs. Epinastine	3.13	2.60 to 3.66	Yes	****				
DMSO vs. Clonidine	0.874	0.345 to 1.40	Yes	****				
DMSO vs. Prazosin	4.59	4.06 to 5.12	Yes	****				
DMSO vs. Amitraz	1.54	1.01 to 2.07	Yes	****				
DMSO vs. Octopamine 5µM	2.33	1.80 to 2.86	Yes	****				
DMSO vs. Octopamine 1.7µM	0.901	0.371 to 1.43	Yes	****				
DMSO vs. Octopamine15µM	1.44	0.913 to 1.97	Yes	****				
Test details	Mean 1	Mean 2	Mean Diff.	SE of diff.	n1	n2	q	DF

DMSO vs. Isoprotenerol	94.9	97.6	-2.77	0.195	2400	2400	14.2	26389
DMSO vs. Tyramine	94.9	88.7	6.16	0.195	2400	2400	31.6	26389
DMSO vs. Salbutamol	94.9	89.4	5.49	0.195	2400	2400	28.2	26389
DMSO vs. Epinastine	94.9	91.7	3.13	0.195	2400	2400	16.1	26389
DMSO vs. Clonidine	94.9	94	0.874	0.195	2400	2400	4.49	26389
DMSO vs. Prazosin	94.9	90.3	4.59	0.195	2400	2400	23.6	26389
DMSO vs. Amitraz	94.9	93.3	1.54	0.195	2400	2400	7.9	26389
DMSO vs. Octopamine 5µM	94.9	92.5	2.33	0.195	2400	2400	12	26389
DMSO vs. Octopamine 1.7µM	94.9	94	0.901	0.195	2400	2400	4.62	26389
DMSO vs. Octopamine15µM	94.9	93.4	1.44	0.195	2400	2400	7.4	26389

S2-ANOVA muscle tone

Muscle tone								
ANOVA summary								
F	1490							
P value	< 0.0001							
P value summary	****							
Are differences among means statistically significant? (P < 0.05)	Yes							
R square	0.531							

Brown-Forsythe test									
F (DFn, DFd)	115 (10, 13178)								
P value	< 0.0001								
P value summary	****								
Significantly different standard deviations? (P < 0.05)	Yes								
Bartlett's test									
Bartlett's statistic (corrected)	5274								
P value	< 0.0001								
P value summary	****								
Significantly different standard deviations? (P < 0.05)	Yes								
ANOVA table	SS	DF	MS	F (DFn, DFd)	P value				
Treatment (between columns)	59736	10	5974	F (10, 13178) = 1490	P < 0.0001				
Residual (within columns)	52843	13178	4.01						
Total	112579	13188							
Data summary									
Number of treatments (columns)	11								
Number of values (total)	13189								
Number of families	1								
Number of comparisons per family	10								
Alpha	0.05								
Dunnett's multiple comparisons test	Mean Diff.	95% CI of diff.	Significant?	Summary					

DMSO vs. Isoprotenerol	-3.55	-3.77 to -3.32	Yes	****				
DMSO vs. Tyramine	1.15	0.925 to 1.37	Yes	****				
DMSO vs. Salbutamol	3.22	3.00 to 3.44	Yes	****				
DMSO vs. Epinastine	1.83	1.61 to 2.06	Yes	****				
DMSO vs. Clonidine	-0.806	-1.03 to -0.584	Yes	****				
DMSO vs. Prazosin	3.32	3.10 to 3.54	Yes	****				
DMSO vs. Amitraz	0.624	0.402 to 0.846	Yes	****				
DMSO vs. Octopamine 5µM	-0.522	-0.744 to -0.300	Yes	****				
DMSO vs. Octopamine 1.7µM	0.102	-0.120 to 0.324	No	ns				
DMSO vs. Octopamine15µM	-3.21	-3.43 to -2.99	Yes	****				
Test details	Mean 1	Mean 2	Mean Diff.	SE of diff.	n1	n2	q	DF
DMSO vs. Isoprotenerol	98.9	102	-3.55	0.0818	1199	1199	43.3	13178
DMSO vs. Tyramine	98.9	97.7	1.15	0.0818	1199	1199	14	13178
DMSO vs. Salbutamol	98.9	95.6	3.22	0.0818	1199	1199	39.3	13178
DMSO vs. Epinastine	98.9	97	1.83	0.0818	1199	1199	22.4	13178
DMSO vs. Clonidine	98.9	99.7	-0.806	0.0818	1199	1199	9.85	13178
DMSO vs. Prazosin	98.9	95.5	3.32	0.0818	1199	1199	40.6	13178
DMSO vs. Amitraz	98.9	98.2	0.624	0.0818	1199	1199	7.63	13178
DMSO vs. Octopamine 5µM	98.9	99.4	-0.522	0.0818	1199	1199	6.38	13178
DMSO vs. Octopamine 1.7µM	98.9	98.8	0.102	0.0818	1199	1199	1.25	13178
DMSO vs. Octopamine15µM	98.9	102	-3.21	0.0818	1199	1199	39.2	13178

S3-ANOVA muscle contraction

Muscle contraction								
ANOVA summary								
F	1330							
P value	< 0.0001							
P value summary	****							
Are differences among means statistically significant? (P < 0.05)	Yes							
R square	0.504							
Brown-Forsythe test								
F (DFn, DFd)	18.8 (11, 14400)							
P value	< 0.0001							
P value summary	****							
Significantly different standard deviations? (P < 0.05)	Yes							
Bartlett's test								
Bartlett's statistic (corrected)	971							
P value	< 0.0001							
P value summary	****							
Significantly different standard deviations? (P < 0.05)	Yes							

ANOVA table	SS	DF	MS	F (DFn, DFd)	P value			
Treatment (between columns)	172621	11	15693	F (11, 14400) = 1330	P < 0.0001			
Residual (within columns)	169895	14400	11.8					
Total	342516	14411						
Data summary								
Number of treatments (columns)	11							
Number of values (total)	13189							
Number of families	1							
Number of comparisons per family	10							
Alpha	0.05							
Dunnett's multiple comparisons test	Mean Diff.	95% CI of diff.	Significant?	Summary				
DMSO vs. Isoprotenerol	-1.99	-2.38 to -1.61	Yes	****				
DMSO vs. Tyramine	11.2	10.8 to 11.5	Yes	****				
DMSO vs. Salbutamol	7.75	7.37 to 8.14	Yes	****				
DMSO vs. Epinastine	4.42	4.03 to 4.80	Yes	****				
DMSO vs. Clonidine	2.55	2.17 to 2.94	Yes	****				
DMSO vs. Prazosin	5.85	5.47 to 6.24	Yes	****				
DMSO vs. Amitraz	2.45	2.07 to 2.84	Yes	****				
DMSO vs. Octopamine 5µM	5.17	4.79 to 5.56	Yes	****				
DMSO vs. Octopamine 1.7µM	1.7	1.31 to 2.08	Yes	****				
DMSO vs. Octopamine15µM	6.09	5.70 to 6.47	Yes	****				

Test details	Mean 1	Mean 2	Mean Diff.	SE of diff.	n1	n2	q	DF
DMSO vs. Isoprotenerol	90.9	92.9	-1.99	0.14	1201	1201	14.2	14400
DMSO vs. Tyramine	90.9	79.7	11.2	0.14	1201	1201	79.6	14400
DMSO vs. Salbutamol	90.9	83.1	7.75	0.14	1201	1201	55.3	14400
DMSO vs. Epinastine	90.9	86.4	4.42	0.14	1201	1201	31.5	14400
DMSO vs. Clonidine	90.9	88.3	2.55	0.14	1201	1201	18.2	14400
DMSO vs. Prazosin	90.9	85	5.85	0.14	1201	1201	41.8	14400
DMSO vs. Amitraz	90.9	88.4	2.45	0.14	1201	1201	17.5	14400
DMSO vs. Octopamine 5µM	90.9	85.7	5.17	0.14	1201	1201	36.9	14400
DMSO vs. Octopamine 1.7µM	90.9	89.2	1.7	0.14	1201	1201	12.1	14400
DMSO vs. Octopamine15µM	90.9	84.8	6.09	0.14	1201	1201	43.4	14400

S4-ANOVA effect of octopamine concentration

Number of families	1							
Number of comparisons per family	3							
Alpha	0.05							
Tukey's multiple comparisons test	Mean Diff.	95% CI of diff.	Significant?	Summary				
Octopamine 5µM vs. Octopamine 1.7µM	-1.43	-1.94 to -0.916	Yes	****				
Octopamine 5µM vs. Octopamine15µM	-0.886	-1.40 to -0.374	Yes	***				
Octopamine 1.7µM vs. Octopamine15µM	0.542	0.0297 to 1.05	Yes	*				
Test details	Mean 1	Mean 2	Mean Diff.	SE of diff.	n1	n2	q	DF
Octopamine 5µM vs. Octopamine 1.7µM	92.5	94	-1.43	0.218	2400	2400	9.25	7197
Octopamine 5µM vs. Octopamine15µM	92.5	93.4	-0.886	0.218	2400	2400	5.74	7197
Octopamine 1.7µM vs. Octopamine15µM	94	93.4	0.542	0.218	2400	2400	3.51	7197
